# Giant esophageal diverticulum with stenosis treated with mediastinoscopic esophagectomy: A case report

**DOI:** 10.1016/j.ijscr.2020.06.047

**Published:** 2020-06-13

**Authors:** Toshikatsu Tsuji, Hiroshi Saito, Kengo Hayashi, Shinichi Kadoya, Hiroyuki Bando

**Affiliations:** Department of Gastroenterological Surgery, Ishikawa Prefectural Central Hospital, 2-1 Kuratsukihigashi, Kanazawa, Ishikawa, 9208530, Japan

**Keywords:** CT, computed tomography, COPD, chronic obstructive pulmonary disorder, PEG, percutaneous endoscopic gastrostomy, Esophageal diverticulum, False diverticulum, Esophageal structural stenosis, Mediastinoscopic esophagectomy

## Abstract

**Introduction:**

An esophageal diverticulum is a rare condition, and surgery is indicated if symptomatic.

We successfully performed mediastinoscopic esophagectomy for a giant esophageal diverticulum with stenosis.

**Presentation of case:**

A 63-year-old man visited our hospital because of dysphagia. He had been pointed out an esophageal diverticulum at a local hospital 13 years before visiting our hospital. Upper gastrointestinal endoscopy revealed an esophageal diverticulum at the lower thoracic esophagus and the structural stenosis in the anal side of the diverticulum. Computed tomography showed a 54 mm esophageal diverticulum at the lower thoracic esophagus. Esophagectomy was required because of the structural stenosis. His medical history included chronic obstructive pulmonary disorder. So, we chose the mediastinal approach to avoid a respiratory complication. We performed mediastinoscopic esophagectomy and esophagogastrostomy via the retrosternal route. The postoperative course was good. At 9 months postoperation, there were no symptoms.

**Discussion:**

Recently, laparoscopic diverticulectomy with myotomy and fundoplication has been considered the best approach in most cases. In the case with the structural stenosis, esophagectomy may be required.

**Conclusion:**

Mediastinoscopic esophagectomy for the patient with poor respiratory function and who need esophagectomy could be an effective and noninvasive candidate procedure.

## Highlights

• An esophageal diverticulum is a rare condition.
• Laparoscopic diverticulectomy with myotomy and fundoplication for an esophageal diverticulum is the common approach.
• Mediastinoscopic esophagectomy for the patient who need esophagectomy could be an effective and noninvasive candidate procedure.

## 1 Introduction

An esophageal diverticulum is a rare condition. Almost all esophageal diverticula are acquired false diverticula. The treatment of esophageal diverticula is generally indicated in symptomatic patients [1]. However, surgical treatment is accompanied by perioperative morbidity. Therefore, it is important to select patients carefully.

Here, we present a case of a giant esophageal diverticulum with esophageal stenosis successfully treated with mediastinoscopic esophagectomy.

## 2 Presentation of case

**Fig. 1:**
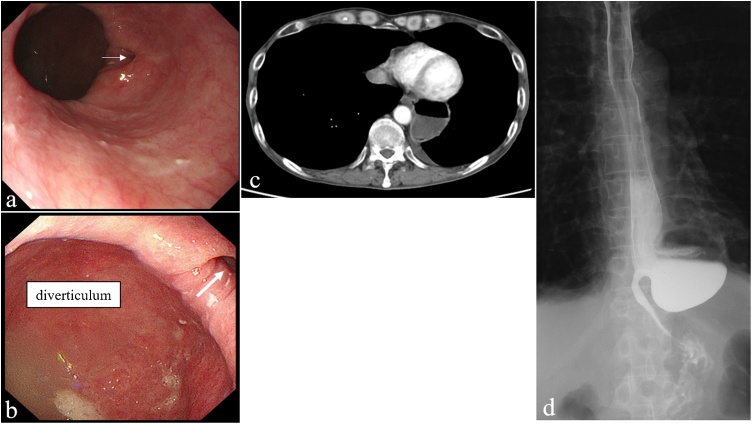
Preoperative imaging findings. a, b: Upper gastrointestinal endoscopy findings. There was a giant esophageal diverticulum at the lower thoracic esophagus and stenosis in the anal side of the diverticulum (arrow). c: CT findings. There was a 54 mm esophageal diverticulum at the lower thoracic esophagus and fluid collection within the diverticulum. d: Upper gastrointestinal contrast study findings. The entrance of the diverticulum was located on the left side of the lower esophagus, and pooling of gastrografin in the diverticulum and delayed passage into the stomach were observed.

**Fig. 2:**
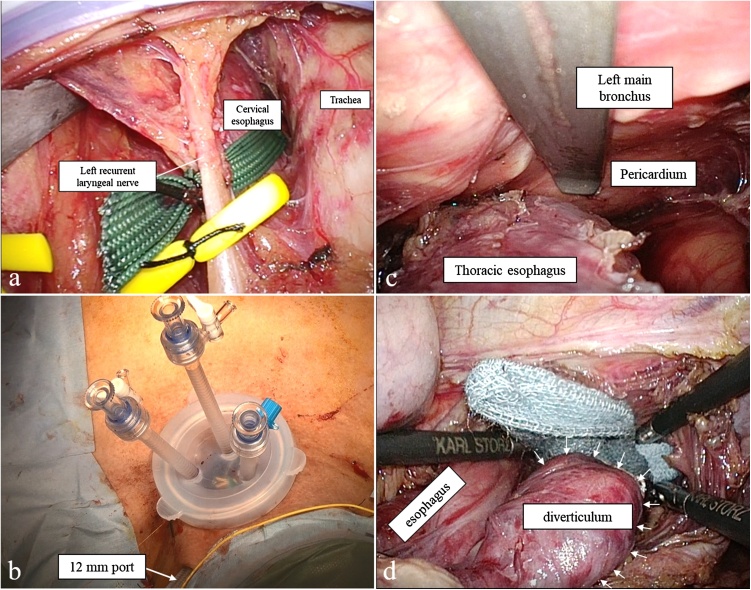
Operative findings. a: The cervical esophagus and left recurrent laryngeal nerve were identified and encircled with tape. b: Port placement. Three 5 mm trocars were inserted through the EZ Access device, and a 12 mm port was added. c: Peeling off the thoracic esophagus under mediastinoscopy. d: The diverticulum was identified on the left side of the lower part of the esophagus (arrows).

A 63-year-old man visited our hospital because of dysphagia. He had pointed out an esophageal diverticulum at a local hospital 13 years before visiting our hospital. However, he did not have reexamination because of no symptom. His medical history included chronic obstructive pulmonary disorder (COPD) (FEV_1.0_%: 59.0 %). Upper gastrointestinal endoscopy revealed a giant esophageal diverticulum at the lower thoracic esophagus and the structural stenosis in the anal side of the diverticulum (Fig. 1a, b). It was impossible to pass through the stenosis with a normal-sized scope. Computed tomography (CT) showed a 54 mm esophageal diverticulum at the lower thoracic esophagus and fluid collection within the diverticulum (Fig. 1c). An upper gastrointestinal contrast study showed that the entrances of the diverticulum were located in the lower esophagus, and pooling of gastrografin in the diverticulum and stenosis in the anal side of the diverticulum were observed (Fig. 1d). Esophagectomy was required because of the structural stenosis. We chose the mediastinal approach to avoid a respiratory complication. Prior to the surgery, percutaneous endoscopic gastrostomy (PEG) was performed because of poor nutrition. We performed the surgery 3 months after PEG. First, the patient was placed in the supine position with the head turned to the right. A 7 cm incision was made 1.5 cm above the left clavicle. After creating a skin flap, the left anterior cervical muscle was removed. The esophagus was dissected from the surrounding tissue and taped (Fig. 2a). The left recurrent laryngeal nerve was identified and encircled by a piece of rubber tape to avoid inadvertent injury (Fig. 2a). Then, a device used for single-port laparoscopic surgery was placed. We usually use a Lap-Protector (Hakko Co., Ltd., Nagano, Japan) for wound protection and an EZ Access device (Hakko Co., Ltd., Nagano, Japan). Three 5 mm trocars were inserted through the EZ Access device, and a 12 mm port was added to use AIRSEAL® intelligent Flow System (Medical Leaders., Ltd., Tokyo, Japan) (Fig. 2b). A scope was 5 mm ENDOEYE flexible scope (LTF-S190−5, Olympus, Tokyo, Japan) and the pneumomediastinum pressure was maintained at 8 mmHg. We dissected along the thoracic esophagus to the pericardium (Fig. 2c). Subsequently, an abdominal procedure was performed. Five ports (two 12-mm ports and three 5-mm ports) were placed. When spreading the esophageal hiatus and pulling the esophagus to the caudal side, the diverticulum was identified on the left side of the lower part of the esophagus (Fig. 2d). The diverticulum was firmly attached to the left pleura and pericardium. The esophagus was completely dissected and connected to the exfoliation layer of the neck. The cervical esophagus was transected, and the specimen was removed from the incision of the upper abdomen. The gastric conduit was constructed, and esophagogastrostomy was performed using a circular stapler via the retrosternal route. The operation lasted 342 min, and the amount of blood loss was 55 ml.

The postoperative course was good, and he had no complication such as the recurrent laryngeal nerve palsy, pneumonia and anastomotic leakage. The drainage tube was removed on postoperative day 4. The oral intake started on postoperative day 7. Since he wanted to be transferred to another hospital for a rehabilitation, he was discharged on postoperative day 38.

An upper gastrointestinal contrast study demonstrated good passage and no stenosis. At 9 months after the operation, there were no symptoms.

**Fig. 3:**
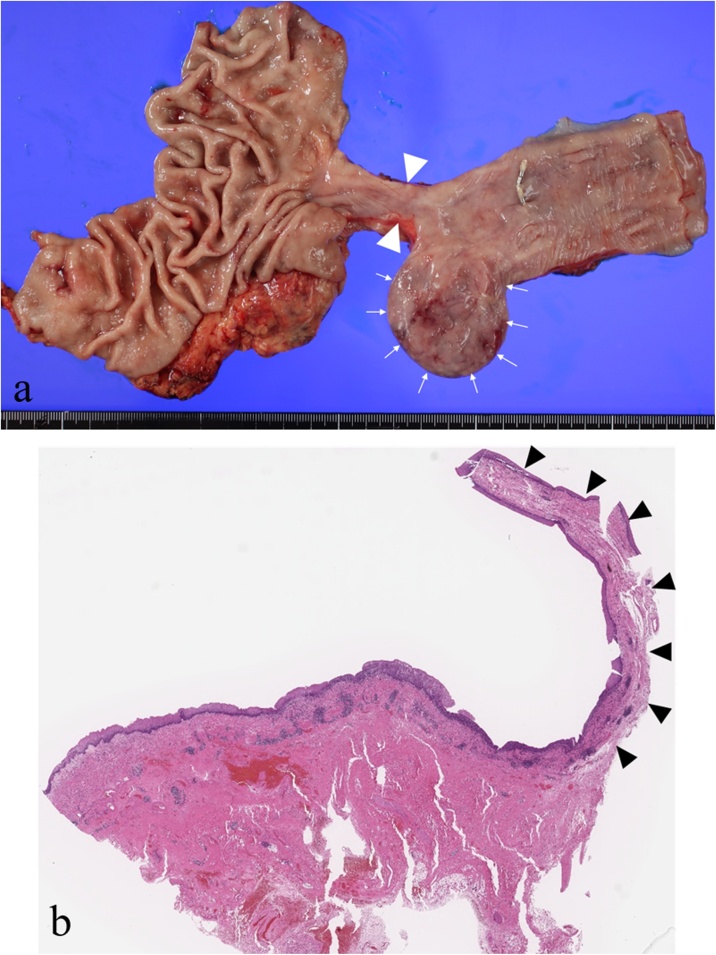
Histopathological findings. a: A 35 × 28 × 25 mm esophageal diverticulum (arrows). Stenosis in the anal side was observed (arrowheads). b: There was no muscularis propria in the diverticulum (arrowheads).

A histopathological examination showed a 35 × 28 × 25 mm esophageal diverticulum with stenosis (Fig. 3a) and without muscularis propria (Fig. 3b).

## 3 Discussion

We successfully performed mediastinoscopic esophagectomy for a giant esophageal diverticulum with the structural stenosis. In this case, we had to perform esophagectomy, not diverticulectomy, because there was the structural stenosis. We chose the mediastinal approach because avoiding a respiratory complication for the patient with COPD. To the best of our knowledge, this is the first report of mediastinoscopic esophagectomy use for an esophageal diverticulum.

An esophageal diverticulum is a rare condition. Diverticula of the middle and lower third of the esophagus are commonly associated with esophageal motility disorders, such as achalasia and achalasia-related diseases [1]. Esophageal diverticula are classified by location: phrenoesophageal (Zenker's diverticulum −70 %), thoracic and mediastinal (10 %), and epiphrenic (20 %) [2]. Most esophageal diverticula are acquired false diverticula that consist of the mucosal and submucosal esophageal layers. Our case is consistent with this description. The most common symptoms are dysphagia, regurgitation, thoracic pain, and pulmonary manifestations related to aspiration. Diverticula should not be treated unless they are symptomatic [2]. Surgery is the standard treatment for a diverticulum and includes diverticulectomy, myotomy, and fundoplication [3]. Some reports have indicated that leakage occurs in 7.7–27.2 % of patients after diverticulectomy and myotomy for esophageal diverticula [[4], [5], [6]]. Recently, laparoscopic diverticulectomy with myotomy and fundoplication has been considered the best approach in most cases [[7], [8], [9]]. Our case was a giant diverticulum with stenosis in the anal side. There was no evidence that the cause of the stenosis was motility disorders because intra-esophageal pressure measurement was not performed. However, we suspected the structural stenosis because there were no characteristic findings such as esophageal rosette in the upper gastrointestinal endoscopy (Fig. 1a, b). So, we planned to perform esophagectomy, not diverticulectomy with myotomy. A laparoscopic approach may be possible in terms of lower esophagectomy, but the mediastinal anastomosis is difficult to perform and increase the incidence of the anastomotic leakage. Therefore, we performed mediastinoscopic esophagectomy and cervical anastomosis. Transhiatal esophagectomy under a mediastinoscope was reported in 1997 by Bumm R et al. [10]. It has been reported that mediastinoscopic esophagectomy is minimally invasive and has fewer complications than thoracotomy; however, knowledge of the mediastinum anatomy is necessary. Single-port mediastinoscopic esophagectomy for esophageal cancer has been reported to be safe and feasible [11,12]. In our procedure, a 12 mm port was added to use AIRSEAL® intelligent Flow System which is a good smoke evacuation system, provides a good field of view, and maintains pressure well.

The postoperative course was good and without complication such as the recurrent laryngeal nerve palsy, pneumonia and anastomotic leakage.

## 4 Conclusion

Laparoscopic diverticulectomy with myotomy and fundoplication for an esophageal diverticulum is the common approach. While Mediastinoscopic esophagectomy for the patient with poor respiratory function and who need esophagectomy could be an effective and noninvasive candidate procedure.

## Declaration of Competing Interest

The authors declare that they have no competing interests.

## Sources of funding

The authors declare that they received no funding support for this report.

## Ethical approval

Ethical approval for this report has been exempted by our institution.

## Consent

Consent to publish was obtained from this patient, and the identity of this patient was protected.

## Author contribution

TT is the first author and prepared the manuscript under the supervision of SK and HB.

TT and SK performed the surgery. HS and KH performed perioperative therapy.

## Registration of research studies

Not applicable.

## Guarantor

Toshikatsu Tsuji, corresponding author of this article.

## Provenance and peer review

Not commissioned, externally peer-reviewed.
